# Highly prevalent at-risk sexual behaviours among out-of-school youths in urban Cameroon

**DOI:** 10.11604/pamj.2018.30.254.15775

**Published:** 2018-08-06

**Authors:** Elvis Enowbeyang Tarkang, Lilian Belole Pencille, Emana Dadah, Melanie Mbungo Nzegge, Joyce Komesuor

**Affiliations:** 1 HIV/AIDS Prevention Research Network, Cameroon (HIVPREC), PO Box 36 Kumba, South-West Region, Cameroon; 2 School of Public Health, University of Health and Allied Sciences, PMB 31 Ho, Volta Region, Ghana

**Keywords:** HIV/AIDS, out-of-school youths, socio-demographic characteristics, sexual risk behavior, urban Cameroon

## Abstract

**Introduction:**

Cameroon has a high prevalence of out-of-school youths. Therefore, research relating to out-of-school youths and HIV/AIDS is imperative, since they might engage in high risk sexual behaviours. The current study investigated the highly prevalent at-risk sexual behaviours among out-of-school youths in urban Cameroon.

**Methods:**

A cross-sectional design was adopted using a self-administered questionnaire to collect data from a cluster sample of 405 out-of-school youths, aged 15-24 years. Statistics was calculated using SPSS version 20 at the level 0.05.

**Results:**

By age less than 16 years, more females, 90.2% than males, 71.8% had experienced sex (p < 0.001); more females, 40.4% than males, 23.2% used condoms during first sex (p < 0.01); more males, 70.8% than females, 46.0% had multiple sequential sexual partners during the last one year prior to this study (p < 0.001); more males, 42.6% than females, 18.0% had multiple concurrent sexual partners during the study period (p < 0.01); more youths who did not belong to a well-defined social network, 80.8% had experienced sex than those who belonged, 55.8% (p < 0.001); more youths who did not belong to a well-defined social network had multiple sequential sexual partners, 46.7% than those who belonged, 32.3% (p < 0.01); more youths who belonged to a well-defined social network, 24.3% used condoms consistently than those who did not belong, 15.4% (p < 0.01).

**Conclusion:**

Sexual risk behaviours exist among out-of-school youths in urban Cameroon. There is need for campaigns and interventions to bring about sexual behaviour change especially among those with low socioeconomic status. Youths should be encouraged to join well-defined social networks.

## Introduction

One of the current challenges in the prevention and control of HIV/AIDS faced worldwide is among youths aged 15 to 24. In sub-Saharan Africa (SSA) more than elsewhere, young people within this age bracket remain the most threatened, accounting for half of all new HIV infections [1]. Therefore, monitoring the sexual behaviours of this vulnerable age group is necessary in order to control the HIV/AIDS pandemic. Youths in Cameroon aged 15-24, comprise 21.5% of the total population [2] and the estimated HIV/AIDS prevalence rate in this group was 2.9% [3]. In fact, these youths are the future of Cameroon and thus, an important age group for the growth and prosperity of the country. Cameroon has a high prevalence of out-of-school youths, representing 56.5% of the total youth population; slightly above 10.2% of these out-of-school youths lack any education whatsoever [4]. Despite this evidence, this population has been hitherto neglected and most studies and interventions in Cameroon have targeted in-school youth [5-7]. In-school youths are easier to reach, making research and interventions cheaper and less complex in terms of logistics. Given the established role of behavioural change in countering the HIV/AIDS pandemic in SSA [6-9], disregarding the out-of-school adolescents is no longer an option. Therefore, research relating to out-of-school youths and young adults and HIV/AIDS in Cameroon seems timely.

In Cameroon, HIV transmission mainly occurs through sexual contact, with 90% of infections due to heterosexual intercourse [10]. Sexual risk behaviours include engaging in unprotected penile-vaginal intercourse, early sexual debut and having multiple sexual partners [11, 12]. These risky sexual practices are influenced by many factors including the lack of accurate information on the modes of transmission of HIV/AIDS, economic conditions, gender inequalities, living place, religion, well-defined social network affiliation, perception of risk of HIV infection, level of education and age [13-17]. Despite a wealth of research on youth, little research has been done on the sexual risk behaviours of out-of-school youths in Cameroon. Thus, this study aims to investigate the highly prevalent at-risk sexual behaviours among out-of-school youths in urban Cameroon. The United Nations (UN) defines youth as those persons between the ages of 15 and 24 [18], therefore for the purpose of the current study, youth will refer to adolescents and young adults between the ages of 15 and 24 years.

## Methods

**Study design:** This study was a population-based cross-sectional survey, using a self-administered questionnaire to collect data. It was conducted in July 2013 in Kumba, the economic capital of the South-West region of Cameroon, which ranks third in the HIV/AIDS prevalence.

**Study site:** Kumba is the administrative headquarters of Meme division, and the economic capital of the Southwest region, thus making it one of Cameroon's wealthiest urban centres, which together with the availability of economic and social amenities, industries and political institutions, has resulted in a high population density [19]. With a total land area of 188.4 Km^2^, the total population of Kumba, a mixture of Christians and Muslims, was estimated at 166,000 inhabitants (51.2% males & 48.8% females) [20], the majority of whom are farmers and traders [21]. Administratively, the city is divided into three local government areas.

**Study population and sampling:** The study population included out-of-school youths in the city of Kumba, who were between the ages of 15 and 24 years and who had dropped out of school either primary or secondary school, or who had never attended at least primary school before but were under apprenticeship learning a trade (hairdressing, tailoring, auto mechanic). Therefore, they depended on their parents/guardians financially. A multistage probability sampling method was applied in this study. A list of all the wards (quarters) in all the three municipalities (local government areas) of Kumba (Kumba I, Kumba II & Kumba III) was used as the sampling frame, to randomly select 15 quarters. Out of these 15 quarters, an average of 27 households per quarter was randomly selected to participate in the study. A list of all out-of-school youths was made and stratified by gender. Proportional sampling according to the population distribution of Kumba was used to select the number of eligible males and females to participate in this study. Within the households, one study participant was selected using simple random sampling. This was done to minimise bias, so that the results obtained could be inferred to the entire study population. Any household without an out-of-school youth was skipped and the next was considered according to the already predetermined order. A written consent was obtained after the potential participants and their parents/guardians (for those below 18 years) were informed of the study's objectives. Only youths aged 15-24 who gave consent to participate and those below 18 years whose parents/guardians gave consent, were included in the study. However, no respondent declined participation in the study, and they participated on a voluntary basis with no financial incentives provided. All the parents/guardians were given the opportunity to withhold or withdraw their children from the study at any time they felt like, but no parent/guardian withdrew or withheld their children from participating.

**Sample size:** The sample size was calculated using Epi Info 6.0 statistical software. The result of a previous study conducted in Nigeria, which showed condom use among out-of-school youths to be 29.0%, was used to calculate the sample size for this study [**22**]. To detect a 10% difference in the rate of condom use with 95% confidence interval (CI) and 80% power, a sample of 368 was needed. With the addition of a 10% nonresponse rate, the final sample size was 405.

**Data collection instrument and data collection:** Data for this study were part of the data set of a bigger study that investigated the knowledge of HIV/AIDS and sexual behaviours among out-of-school youths in Kumba in the Southwest region of Cameroon. The reliability of the questionnaire used for this study was tested using the reliability coefficient and by pretesting the questionnaire. The validity was also established by constructing items to represent the different sections of the study topic, based on literature review. The questionnaire was designed as an adaptation from previous studies [7, 9, 23, 24], all adapted from the Center for Disease Control and Surveillance, Youth Risk Behaviour Questionnaire [25], to collect data on socio-demographic characteristics and sexual behaviours. A pretest of the questionnaire was done on a convenience sample of 20 out-of-school youths of both genders who did not take part in the study proper, for clarity and to ascertain internal consistency. Respondents were given the self-administered questionnaires in English, after they were arranged to sit comfortably in the city council conference room. Confidentiality was maintained by providing a private place for the respondents during data collection. Confidentiality was also maintained because only the researcher had access to the completed questionnaires, which were locked up. Subsequent to the acceptance of the research report, these would be destroyed. Four trained research assistants (2 males & 2 females) of the same age group as the participants, assisted those who could not read or write. The completed questionnaires were checked by the research assistants for errors and missing data before participants were allowed to go. Anonymously completed questionnaires were kept in a separate container from the signed informed consent forms in order to maintain anonymity.

**Ethical considerations:** Ethical clearance for this study was obtained from the Institute of Philosophy, Religious and Social Studies of the Cameroon Christian University (CCU) Ethical Review Committee. Approval for this study was obtained from the HIV/AIDS Prevention Research Network, Cameroon (HIVPREC) research and ethics committee and from the Kumba Municipal (local government) authorities.

**Data analysis:** Data were edited, cleaned, coded, entered and analysed using the Statistical Package for Social sciences (SPSS) version 20 software program. Probability (P) values were interpreted at the 0.05 level of significance. This was a technique to minimise error. Data were summarized by means of descriptive statistics including the frequency table. Two-sided chi-square tests for association were computed to detect any associations between sexual behaviours and socio-demographic characteristics of the participants.

**Measures:** Socio-demographic characteristics included: age which was self-reported in years, sex divided into two categories (male and female), marital status categorised into single and others, religion categorised into three groups (Christians, Muslims and others), well-defined social network affiliation was divided into (yes or no) and fathers' and mothers' monthly incomes, categorised into two groups (more than 200 000XAF and 200 000XAF or less). Sexual experience was categorised into 1 = yes and 0 = no. Condom use during first sexual intercourse categorised into two groups (1 = yes, 0 = no), condom use during last sexual encounter was categorised into 1 = yes and 0 = no, regularity of condom use during sexual intercourse was divided into four categories (1 = always, 2 = most of the time, 3 = seldom and 4 = never). These questions were asked only to respondents who were sexually active. The coefficient alpha for the 3-item condom use scale was 0.80. Age of first sex was self-reported in years, number of sexual partners in the last one year prior to this study and during the period of this study was categorised into more than one and one or less. The alpha reliability for the 2-item number of sexual partners scale was 0.88.

## Results

**Socio-demographic characteristics:** The socio-demographic characteristics of the study population are provided in Table 1. There was a total of 208 male and 197 female out-of-school youths. All were between the ages of 15 and 24 years, (mean age (SD) was 18.94 (2.11)), with the majority, 93.1% being single. Most of them, 94.6% were Christians and 88.9% belonged to a social group (social club, sports club or church youth group). The majority, 64.6% indicated that their fathers' monthly incomes were less than 200 000XAF (US$ 13.00 a day) and 81.6% indicated that their mothers' monthly incomes were less than 200 000XAF (US$ 13.00 a day) (Table 1).

**Table 1:** Socio-demographic characteristics of out-of-school youths in an urban area of Cameroon

Characteristics	Frequency	Percentage
**Age Group (n=405)**		
15-24	405	100.0
**Gender (n=405)**		
Male	208	51.4
Female	197	48.6
**Marital Status (n=405)**		
Single	375	93.1
Others	28	6.9
**Religious Affiliation (n=404)**		
Christian	382	94.6
Muslim	22	5.4
Others	0	0.0
**Social network affiliation (n=397)**		
Yes	353	88.9
No	44	11.1
**Father’s monthly income (n=395)**		
200 000XAF and above	140	35.4
Less than 200 000XAF	255	64.6
**Mother’s monthly income (n=399)**		
200 000XAF and above	74	18.5
Less than 200 000XAF	325	81.5

**Sexual behaviours of out-of-school youths:** Table 2 shows the percentage distribution of sexual behaviours of the out-of-school youths in this study. The majority, 55.6% had experienced sexual intercourse, with the majority, 80.4% having experienced sexual intercourse by age 16 years. A high proportion of the sexually experienced out-of-school youths in this study, 57.3% reported multiple sexual partners in the previous one year, while a significant proportion, 27.7% reported multiple concurrent sexual partners during the period of this study. The proportion of sexually experienced out-of-school youths in the current study who used condoms during their first and last sexual encounters was low, 29.8% and 48.9% respectively. In the same vein, the proportion who used condoms consistently during sexual intercourse was low, 18.7%.

**Table 2:** Sexual behaviours of out-of-school youths in an urban area of Cameroon

Sexual behaviours	Frequency	Percentage
**Ever had sexual intercourse with a male/female partner (n=405)**		
Yes	225	55.6
No	180	44.4
**Age at which first sexual intercourse occurred (n=225)**		
16 years or less	181	80.4
More than 16 years	44	19.6
**Number of sexual partners in the past one year (n=225)**		
One or less	96	42.7
More than one	129	57.3
**Number of concurrent sexual partners at present (n=224)**		
One or less	162	72.3
More than one	62	27.7
**Condom use during first sexual encounter (n=225)**		
Yes	67	29.8
No	158	70.2
**Condom use during last sexual encounter (n=225)**		
Yes	110	48.9
No	115	51.1
**Regularity of condom use during sexual intercourse (n=225)**		
Always	42	18.7
Most of the time	74	32.9
Seldom	39	17.3
Never	70	31.1

**Associations between sexual behaviours and gender:** Table 3 shows the associations between sexual behaviours and gender among sexually active out-of-school youths. By age less than 16 years, more females than males had experienced sexual intercourse (X^2^ = 46.03; p = 0.000). In the same vein, more females than males used condoms during their first sexual encounters (X^2^=9.97; p=0.007). However, more males than females had multiple sexual partners either sequentially or concurrently (X^2^ = 19.84; p = 0.000) and X^2^ = 15.31; p = 0.002) respectively.

**Table 3:** Associations between sexual risk behaviours and gender

Sexual risk behaviours	Gender		X^2^	P-values
	Male	Female		
First sexual intercourse by 16 years	89 (71.8%)	92 (90.2%)	46.03	0.000
Multiple sexual partners in the past one year before this study	85 (70.8%)	46 (46.0%)	19.84	0.000
Multiple concurrent sexual partners during the period of this study	46 (42.6%)	16 (18.0%)	15.31	0.002
Condom use at first sexual encounter	29 (23.2%)	42 (40.4%)	9.97	0.007

**Associations between sexual risk behaviours and social network affiliations:** As illustrated in Table 4, more out-of-school youths who did not belong to any well-defined social network had experienced sexual intercourse and had multiple sexual partners in the previous one year than those who belonged to a well-defined social network (X^2^ = 22.75; p = 0.001) and (X^2^ = 35.75; p = 0.008) respectively. On the contrary, more sexually experienced out-of-school youths who belonged to a social group used condoms during first sex, during last sex and consistently than those who did not belong to any social group (X^2^ = 22.81; p = 0.029), (X^2^ = 29.32; p = 0.003) and (X^2^ = 44.43; p = 0.007) respectively.

**Table 4:** Associations between sexual risk behaviours and well-defined social network affiliation

Sexual risk behaviours	Social network affiliation		X^2^	P-values
	Yes	None		
Having experienced sexual intercourse	197 (55.8%)	12 (80.0%)	22.75	0.001
Multiple sexual partners in the past one year	114 (32.3%)	7 (46.7%)	35.75	0.008
Condom use at first sexual intercourse	32 (31.4%)	3 (25.0%)	22.81	0.029
Condom use at last sexual intercourse	60 (58.8%)	7 (53.8%)	29.32	0.003
Consistent condom use during sexual intercourse	25 (24.3%)	2 (15.4%)	44.33	0.007

**Associations between sexual risk behaviours and economic status:** More out-of-school youths whose mothers' monthly incomes were less than 200 000XAF (US$ 13.00 a day) (57.8%) had experienced sexual intercourse than those whose mothers' monthly incomes were more than 200 000XAF (45.9%) (X^2^ = 9.48; p = 0.024).

## Discussion

This study investigated the highly prevalent at-risk sexual behaviours among out-of-school youths in urban Cameroon. Sexual risk behaviours include engaging in unprotected penile-vaginal intercourse, early sexual debut and having multiple sexual partners [11, 12]. Majority of the sexually active out-of-school youths (80.4%) had experienced sex by age less than 16 years. This rate is higher than that obtained among in-school youths in urban Cameroon, 60.2% [6]. This discrepancy may have resulted from the fact that out-of-school youths are less exposed to formalised HIV/AIDS information and sexuality education than their in-school peers. Knowing the age at which the respondents first had sexual intercourse is important as it could indicate at what age sex education, as well as condom knowledge, should be provided. Early sexual debut can place out-of-school youths at increased risk of unintended pregnancy, HIV/AIDS infection and other STIs. Youths who begin sexual activities early are more likely to have sex with high risk partners and are less likely to use condoms [26]. In the current study, only 18.7% of the sexually active out-of-school youths reported consistent condom usage. There is therefore the need for formalised HIV/AIDS and sexuality education among out-of-school youths in urban Cameroon, with focus on strategies to delay sexual debut and increase safe sexual practices. Overall, 57.3% of the sexually active out-of-school youths in this study mentioned having had more than one sexual partner in the past one year prior to the study and 27.7% mentioned having more than one concurrent sexual partner during the period of this study and these are higher than those obtained among in-school youths, 33.3% and 10.7% respectively [6]. HIV/AIDS and sexuality education among out-of-school youths in Cameroon, with emphasis on strategies to reduce the number of sexual partners and to increase safe sexual practices is imperative. Multiple concurrent sexual partnerships in which condom use tend to be low is among the key drivers to HIV/AIDS infection. HIV infection is more likely to occur within long term multiple concurrent sexual partnerships as people are less likely to consistently use condoms within these more regular relationships [27]. Within a set of serial relationships, transmission is linear, so early partners are protected. In the case of concurrent partners, early partners continue to be at risk as a later partner infects the subject because the partners overlap in time. A significant number of out-of-school youths in this study engage in sexual intercourse with multiple partners without condom use and are therefore exposed to HIV transmission. Educate on sexual risk behaviours and the resultant dangers of such behaviours among out-of-school youths in urban Cameroon is of the essence.

Information was also sought on use of condoms among those who had ever had sex. This was to determine the proportion of the set of youths who were engaging in high-risk sex. The proportion of sexually experienced out-of-school youths in this study who used condoms during their first and last sexual encounters was low, 29.8% and 48.9% respectively. In the same vein, the proportion who used condoms consistently during sexual intercourse was low, 18.7%. These findings are in accordance with those of Fagbamigbe, Adebowale & Olaniyan (2011) [28] among out-of-school youths in Nigeria, but lower than those obtained among in-school youths in Cameroon [9]. These percentages as reported in the current study are quite low, bearing in mind that 60% of the respondents were sexually active. This is a source of concern now that the prevalence of HIV/AIDS is high in Cameroon. The following socio-demographic characteristics: (gender, well-defined social network affiliation and mother's monthly income) were identified in this study as having significant associations with sexual risk behaviours (sexual experience, age of sexual debut, number of sexual partners and condom use) among out-of-school youths in urban, Cameroon [14, 29, 30]. More out-of-school youths whose mothers' monthly incomes were less than 200 000XAF (US$ 13.00 a day), (57.8%) had experienced sexual intercourse than those whose mothers' monthly incomes were more than 200 000XAF, (45.9%). This could be explained by the fact that youth in Cameroon, especially females are more attached to their mothers than their fathers and therefore depend on their mothers financially than their fathers. Therefore, mothers with low monthly income may find it difficult to meet the financial needs of their children, and as a consequence, these children might engage in undesirable sexual intercourse for financial gains in order to meet their needs. In Cameroon, the observed minimum wage is 32 800 XAF per month [31]. This monthly income translates to approximately 1 000 XAF per day (US$ 2.00). According to the World Bank (2009) [32], Cameroon, with a gross national income per capita (GNI) of US$ 1 150.00 is ranked number 158 in the world and is considered to be a low-income country. Low economic status of parents may put out-of-school youths at risk of engaging in undesirable sexual behaviours because these youths might not have the financial means to practise safe sex such as buying and using condoms to prevent HIV/AIDS. Economic hardships could increase youths' high-risk situations such as HIV/AIDS, pregnancies and drug abuse [16].

Many youths therefore come from poverty-stricken homes, with most of their parents on an income of less than 200 000 XAF a month and poverty could be a major setback putting out-of-school youths' health at stake. Poor out-of-school youths might find it difficult to initiate and maintain safer sexual practices such as condom use or being faithful to one partner even if their risk perception of HIV/AIDS is high. Poverty might be an important factor working in favour of increased prevalence of HIV/AIDS among out-of-school youths [33]. Therefore, poor female out-of-school youths may be pushed into sexual risk behaviours especially with older men to ensure survival, receive material goods to relieve poverty; and this behaviour is likely to be associated with increased HIV risk. Low socio-economic status appears to be associated with out-of-school youths having experienced sex, in accordance with [15]. Well-defined social network refers to social youth club, sports group or church youth group. Youths taking part in youth club activities are expected to be more knowledgeable with regard to HIV/AIDS and sexual behaviours since they have opportunities to discuss sexual and HIV/AIDS-related issues with their peers. One could therefore expect that belonging to youth groups could motivate youths to practise safe sex. In accordance with other authors [34, 35], who report that youths who regularly attend church services and church meetings might be less likely to be sexually experienced at younger age, and that participating in sports has a delaying effect on initiation of the first sexual intercourse, lower frequency of sexual intercourse and decrease number of lifetime sexual partners, out-of-school youths in this study who belonged to a well-defined social network, were less likely to engage in sexual risk behaviours than those who did not belong to any social network. In this study, more male than female out-of-school youths engaged in sexual risk behaviours (multiple sexual partners and non-use of condoms) which may expose them to HIV transmission. These findings are in agreement with other studies in Cameroon [3, 16] and Nigeria [36-38], which report higher rates of risky sexual activities among male out-of-school youths. Gender differences, in terms of assumed self-reliance and family freedom by male youths relative to females can be the explanatory factor in this regard. In the African setting, male children often enjoy more freedom than their female counterparts on sexual issues. However, in this study, more females had experienced sex by age less than 16 years than their male counterparts. This could be attributed to the lower socio-economic status of women compared to men, which causes women to sell sex to men, in order to make ends meet.

In most cultures in Africa, including Cameroon, women are expected to be receivers of decisions made by men with regard to sexual behaviours. Females are brought up to be submissive to males especially in matters of sex. Women are discriminated against in terms of employment and education and with increasing levels of poverty in Africa, including Cameroon, women find themselves in casual relationships with men, sometimes older men, who are more likely to be infected than younger males. Women therefore could find it difficult to demand safe sex, as they become dependent mainly on the older men economically [39]. The context of gender inequalities could therefore place women at a greater risk of being infected with HIV/AIDS. There are no accurate global estimates of the prevalence of adolescents aged less than 18 years who sell sex. However, many studies show that substantial percentages of sex workers in many countries began selling sex aged younger than 18 years. In Burkina Faso, 6% of female sex workers were less than 18 years in 2002 [40]. In eight countries in eastern and southern Africa, median HIV prevalence among sex workers younger than 25 years is 11% [41]. The young women who sell sex (YWSS) group is more vulnerable than older cohorts to health harms-including STIs, HIV and violence [41-43]. According the Convention on the Rights of the Child-the most widely ratified human rights treaty [44], international agreements define YWSS under 18 years as victims of trafficking and/or sexual exploitation and making many providers wary of legal repercussions under international laws for sexually exploited children. Whatever, this vulnerable population needs specific health interventions including access to sexual and reproductive health and rights, and HIV treatment, prevention, and care, despite the law and policy barriers and the frequent lack of confidential and adolescent-friendly HIV services.

**Limitations:** The study should be interpreted in light of the following limitation: being a cross-sectional study, it is not possible to draw conclusions about causality of any of the identified associations. Furthermore, given that the study was conducted in one location, it may not be applicable to other settings. Thirdly, self-assessments of sexual behaviours through questionnaires are prone to a number of biases that could affect the validity and reliability of the results. Also, HIV/AIDS and sexual issues are very sensitive and could limit free expression of the out-of-school youths. Assurance of confidentiality of the responses, the presence of research assistants in the room to answer possible questions raised by respondents during data collection, and the simplicity and direct nature of the questions in the questionnaires minimised this effect.

## Conclusion

The findings of this study suggest that out-of-school youths in urban Cameroon have needs in terms of HIV/AIDS education and prevention. All sexuality education programmes and HIV/AIDS education should commence before the age of 15 years. Although condom is generally assumed to be affordable (less than US$ 0.25) and easily available in shops and chemists across Cameroon, intending users may have problems obtaining it as people often look down on condom buyers especially youths as being irresponsible. An important effort should be made in order to inform the general population that condom buyers are, in fact, responsible youths. Strategies for providing HIV/AIDS and sexuality education among out-of-school youths may include organising seminars and workshops targeting out-of-school youths in the urban settings of Cameroon. Also, out-of-school youths should be encouraged to join well-defined social networks. Liberalization of condom in terms of free distribution to youths in urban Cameroon can tremendously fill the gap of sexual risk behaviours among out-of-school youths. However, programs for total abstinence from sex among these out-of-school youths could be a better solution to safe sexual practices. In this regard, sexuality education is essential.

### What is known about this topic

- In sub-Saharan Africa (SSA) more than elsewhere, young people within this age bracket remain the most threatened, accounting for half of all new HIV infections;
- Cameroon has a high prevalence of out-of-school youth, representing 56.5% of the total youth population; slightly above 10.2% of these out-of-school youth lack any education whatsoever; Despite the evidence, this population has been hitherto neglected and most studies and interventions in Cameroon have targeted in-school youth who are easier to reach, making research and interventions cheaper and less complex in terms of logistics. Given the established role of behavioural change in countering the HIV/AIDS pandemic in SSA, disregarding the out-of-school adolescents is no longer an option. Therefore, research relating to out-of-school youths and young adults and HIV/AIDS in Cameroon seems timely;
- In Cameroon, HIV transmission mainly occurs through sexual contact, being 90% of infections due to heterosexual intercourse, and sexual risk behaviours include engaging in unprotected penile-vaginal intercourse, early sexual debut and having multiple sexual partners.

### What this study adds

- More male out-of-school youths in Kumba, Southwest region of Cameroon engaged in sexual risk behaviours than their female counterparts;
- Out-of-school youths who did not belong to any well-defined social network, practised sexual risk behaviours than those who belonged to a social network;
- These findings could health promoters in designing and implementing programmes and strategies to the highly prevalent at-risk sexual behaviours among out-of-school youth in urban Cameroon.

## Competing interests

The authors declare no competing interests.
